# Supplementary material for: Counterfactual relief
Source: Mem Cognit. 2025 Dec 8;54(4):1373–87. doi: 10.3758/s13421-025-01815-9 (PMC13253781; doi:10.3758/s13421-025-01815-9)
Supplement: Supplementary file 1 — Supplementary file1 (DOCX 28 KB) [file 13421_2025_1815_MOESM1_ESM.docx]

Supplemental 1: Study 1 Questionnaire

**Please read the following short description.** We may experience relief when we learn that something bad that could have happened did not happen. We may be anxious before we discover that the bad thing did not happen. For example, a student worried about her exam performance might be anxious before learning that she has passed. Sometimes people might feel relief even when they are not worried before. **We are particularly interested in cases where the person who experiences the relief is not anxious, worried or fearful prior to learning that the bad thing has not happened.** For example, someone might be relieved when they learn that the flight they missed ended up crashing. Or, an employee might be surprised to learn that some of his colleagues lost their jobs last week and feel relieved that he is not in that position.
 **We would like you to take a moment to think about a time in your life when you felt relieved because something bad that could have happened did not happen. This can be any sort of bad thing; it does not need to be about your career or your education. We are interested in whether you can think about a time when, before you felt relief, you were not thinking about a bad event happening, like in the examples above.**

Q1 Have you ever felt relief in circumstances like this? That is, have you felt relieved when you found out that you avoided something bad, but you did not realise in advance that the bad thing might happen.

- Yes (1)
- No (2)

Display This Question:

If Have you ever felt relief in circumstances like this? That is, have you felt relieved when you fo... = No

Q2a That’s fine. We are also interested in other times that people might feel the emotion of relief. Now we would like you to think about a time when you felt relief because something bad that could have happened, did not happen. This could be a time where you were worried about the bad thing happening before you felt relief or it could be a time when you were not worried about a bad thing happening before you felt relief.

Display This Question:

If Have you ever felt relief in circumstances like this? That is, have you felt relieved when you fo... = Yes

Q2b You say you have you felt relieved when you found out that you avoided something bad, but you **did not** realise in advance that the bad thing might happen.

Q3 Please provide a description of the circumstances in which you felt this emotion in the text box below.

________________________________________________________________

________________________________________________________________

________________________________________________________________

________________________________________________________________

________________________________________________________________

Q4
Bearing in mind the event that you have described: ***Insert participant text***Please rate the intensity of the relief that you experienced. Use your mouse, touchpad, or touchscreen to move the red dot along the scale below.

|  | A little | A lot |
| --- | --- | --- |


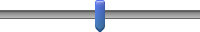


You have described an instance when you felt relieved because something bad that could have happened did not happen. Please answer some other questions about this instance in which you felt relief.

Q5
**What was the bad thing that could have happened?** Provide your answer in the text box below.

________________________________________________________________

________________________________________________________________

________________________________________________________________

________________________________________________________________

________________________________________________________________

Q6 **How bad would the bad thing have been?** Use your mouse, touchpad, or touchscreen to move the red dot along the scale below.

|  | 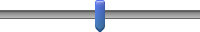Not bad at all | Very bad |
| --- | --- | --- |

Display This Question:

If Have you ever felt relief in circumstances like this? That is, have you felt relieved when you fo... = No

Q7 **Can you confirm that you were thinking about the possibility of the bad thing happening before you felt relief?**

- That's correct. I did realise in advance that the bad thing might happen. (1)
- That's not correct. I did not reliase in advance that the bad thing might happen. (2)

Display This Question:

If Have you ever felt relief in circumstances like this? That is, have you felt relieved when you fo... = Yes

Q8 **Can you confirm that you were not thinking about the possibility of the bad thing happening before you felt relief?**

- That's correct, I did not realise in advance that the bad thing might happen. (1)
- That's not correct. I did realise in advance that the bad thing might happen. (2)

Display This Question:

If Can you confirm that you were thinking about the possibility of the bad thing happening before yo... = That's correct. I did realise in advance that the bad thing might happen.

Or Can you confirm that you were not thinking about the possibility of the bad thing happening befor... = That's not correct. I did realise in advance that the bad thing might happen.

Q9 **Before you felt relief, did you feel anxious, worried, or scared about the bad thing possibly happening?**

- Yes (1)
- No (2)

Display This Question:

If Before you felt relief, did you feel anxious, worried, or scared about the bad thing possibly hap... = Yes

Q10 **You say you felt anxious, worried, or scared before you felt relief. How strongly did you feel that way?** Use your mouse, touchpad, or touchscreen to move the red dot along the scale below.

|  | A little | A lot |
| --- | --- | --- |


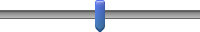


Display This Question:

If Before you felt relief, did you feel anxious, worried, or scared about the bad thing possibly hap... = Yes

Q11 **You say you felt anxious, worried, or scared before you felt relief. Did this feeling stop completely after you learned that the bad thing did not happen?**

- Yes (1)
- No (2)

Display This Question:

If You say you felt anxious, worried, or scared before you felt relief. Did this feeling stop comple... = No

Q12 **You say you still felt anxious, worried, or scared after you learned the bad thing did not happen. How strongly did you feel that way?** Use your mouse, touchpad, or touchscreen to move the red dot along the scale below.

|  | A little | A lot |
| --- | --- | --- |


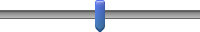


Supplemental 2: Study 2 example question 1 (purely counterfactual relief)

We would like you to take a moment to read about a time when someone felt the emotion of relief because something bad that *could* have happened *did not* happen. The description can be seen below.

*I got out of my dad's car 2 minutes before he was in an accident with himself my brother and my mum in the car. My brother was very lucky as the front seats collapsed and he was thrown under them saving him. If I had been in the car It would have been a different story.*

Q1 Please rate the intensity of the relief you think this individual experienced. Use your mouse, touchpad, or touchscreen to move the red dot along the scale below.

|  | A little | A lot |
| --- | --- | --- |


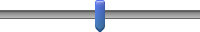


The description of the bad thing that could have happened can be seen below.

*Myself and my brother could have died.*

Q2 How bad would the bad thing have been? Use your mouse, touchpad, or touchscreen to move the red dot along the scale below.

|  | Not bad at all | Very bad |
| --- | --- | --- |


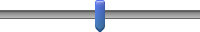


Q3 Have you personally experienced relief in a situation similar to that described?

- Yes (1)
- No (2)

Q4 How best would you describe the topic of the experience you have just read about?

- Education (1)
- Death (2)
- Career (3)
- Romantic relationships (4)
- Parenting (5)
- Lost and missing (6)
- Health (7)
- Finance and legal (8)
- Emergency (9)
- Other (please specify) (10) __________________________________________________

Supplemental 3: Study 2 example question 2 (anxiety cessation relief)

We would like you to take a moment to read about a time when someone felt the emotion of relief because something bad that *could* have happened *did not* happen. The description can be seen below.
*I was pregnant with my first child (after several miscarriages) at the 12 week scan they identified a potential problem. The rest of the pregnancy was very stressful with scans every 2 weeks. If it hadn't been for my consultant who decided to get him out at 34 weeks he would have died. The relief when he was born and everything was perfect other than he was very small was unforgettable.*

Q1 Please rate the intensity of the relief you think this individual experienced. Use your mouse, touchpad, or touchscreen to move the red dot along the scale below.

|  | A little | A lot |
| --- | --- | --- |


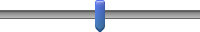


The description of the bad thing that could have happened can be seen below.

*My baby could have died*

Q2 How bad would the bad thing have been? Use your mouse, touchpad, or touchscreen to move the red dot along the scale below.

|  | Not bad at all | Very bad |
| --- | --- | --- |


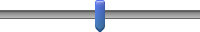


Q3 Have you personally experienced relief in a situation similar to that described?

- Yes (1)
- No (2)

Q4 How best would you describe the topic of the experience you have just read about?

- Education (1)
- Death (2)
- Career (3)
- Romantic relationships (4)
- Parenting (5)
- Lost and missing (6)
- Health (7)
- Finance and legal (8)
- Emergency (9)
- Other (please specify) (10) __________________________________________________

Supplemental 4: Study 3 example purely counterfactual question format

**We would like you to take a moment to read about a time when someone felt the emotion of relief because something bad that *could* have happened *did not* happen. The description can be seen below:**
*I had gone to the dentist for a routine check-up. At the end of the check-up she told me that though she thought that I might have needed a filling, she had double checked and the tooth she was concerned about was fine.*

**Q1 Please rate the intensity of the relief you think this individual experienced. Use your mouse, touchpad, or touchscreen to move the red dot along the scale below.**

|  | A little | A lot |
| --- | --- | --- |


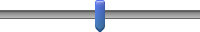


**The description of the bad thing that could have happened can be seen below.**
*Having to get a filling*

**Q2 How bad do you think it would have been if this had happened? Use your mouse, touchpad, or touchscreen to move the red dot along the scale below.**

|  | Not bad at all | Very bad |
| --- | --- | --- |


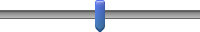


Supplemental 5: Study 3 example anxiety cessation question format

**We would like you to take a moment to read about a time when someone felt the emotion of relief because something bad that *could* have happened *did not* happen. The description can be seen below.**
*I had gone to the dentist for a routine check-up. At the start of her examination the dentist told me that she thought that I might need a filling but would need to double check and I was worried the whole time she was working on me. At the end of the check-up she told me that though she thought that I might have needed a filling, she had double checked and the tooth she was concerned about was fine.*

**Q1 Please rate the intensity of the relief you think this individual experienced. Use your mouse, touchpad, or touchscreen to move the red dot along the scale below.**

|  | A little | A lot |
| --- | --- | --- |


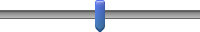


**The description of the bad thing that could have happened can be seen below.**
*Having to get a filling*

**Q2 How bad do you think it would have been if this had happened? Use your mouse, touchpad, or touchscreen to move the red dot along the scale below.**

|  | Not bad at all | Very bad |
| --- | --- | --- |


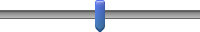


Supplemental 5: Study 3 all versions of vignettes

| Description of Experience (purely counterfactual) | Description of Experience (anxiety cessation) | Bad thing that could have happened |
| --- | --- | --- |
| I had gone to the dentist for a routine check-up. At the end of the check-up she told me that though she thought that I might have needed a filling, she had double checked and the tooth she was concerned about was fine. | I had gone to the dentist for a routine check-up. At the start of her examination the dentist told me that she thought that I might need a filling but would need to double check and I was worried the whole time she was working on me. At the end of the check-up she told me that though she thought that I might have needed a filling, she had double checked and the tooth she was concerned about was fine. | Having to get a filling |
| I was originally due to travel home for Christmas on Christmas Eve. I was able to go home a day early as my work shifts were changed. As my train began to leave the station, I saw that all the trains after mine had been cancelled. | I was originally due to travel home for Christmas on Christmas Eve. Some other trains had been cancelled and I was worried for days that if my train was cancelled, I wouldn’t make it home in time for Christmas. I was able to go home a day early as my work shifts were changed. As my train began to leave the station, I saw that all the trains after mine had been cancelled. | Not making it home in time for Christmas |
| I heard on the news that there was a terrorist attack on Westminster Bridge. When she rang me that evening, my mother told me that her meeting in Westminster that day had gone well and that although she had been right beside the location of the attacks, she had left just before they started. | I heard on the news that there was a terrorist attack on Westminster Bridge. My mother was in London for a meeting in Westminster that day and I was worried all day that she might have been caught up in the attack. When she rang me that evening, my mother told me that her meeting in Westminster that day had gone well and that although she had been right beside the location of the attacks, she had left just before they started. | My mother getting caught up in the terrorist attack |
| At the very last minute, I decided to take a placement year during my degree back in the 1970s. I hadn’t been in touch with any of my classmates whilst on placement, and it was only as I walked into my first lecture after the placement that I realised that I might be the only one who had taken a year out and that I might have no one to talk to for my entire final year. Happily, in the first lecture I attended on my return I immediately saw several faces I recognised from my year group and knew that I’d have people to socialise with. | At the very last minute, I decided to take a placement year during my degree back in the 1970s. I hadn’t been in touch with any of my classmates whilst on placement, and in the last few months of the placement I worried that I might be the only one who had taken a year out and that I would have no one to talk to for my entire final year. Happily, in the first lecture I attended on my return I immediately saw several faces I recognised from my year group and knew that I’d have people to socialise with. | Not having anyone to socialise with. |
| My uncle promised to help me to sort out accommodation before I started university in London. The next day he rang me to tell me that although when he agreed to help he was certain that he would never find a place for me, he’d actually found an apartment within my budget. | My uncle promised to help me to sort out accommodation before I started university in London. I was very worried because when he first promised to help, he said that he was sure he wouldn’t be able to find me accommodation because everywhere in London was so expensive. The next day he rang me to tell me that although when he agreed to help he was certain that he would never find a place for me, he’d actually found an apartment within my budget. | Not being able to find accommodation |
| The company I work for was making plans to make everyone in my group redundant. None of us knew at the time because they hadn’t told anyone that they had been having financial difficulties for months. The company announced it had won a big contract and that, consequently, it wouldn’t have to follow through with its plans to lay people off. | The company I work for was making plans to make everyone in my group redundant. I was worried for months that I might lose my job. The company announced that it has won a big contract and that, consequently, it wouldn’t have to follow through with its plans to lay people off. | Being made redundant |
